# The effect of finding relationships, using variables, drawing diagrams and simplification strategies on the development of formulating skills

**DOI:** 10.3389/fpsyg.2026.1722584

**Published:** 2026-02-03

**Authors:** Hasan Temel

**Affiliations:** Department of Mathematics Education, Necatibey Faculty of Education, Balıkesir University, Balıkesir, Türkiye

**Keywords:** cognitive load theory, drawing diagrams, finding relationships, formulating, mathematics education, metacognition, simplification, using variables

## Abstract

**Background:**

formulation serves as a bridge between real-life situations and mathematics, is a critical skill in processes that are crucial for mathematics education, such as problem solving, mathematical literacy, modeling, realistic mathematics education, conceptual thinking, and STEM education, but also emerges as a skill that must be considered for cognitive processes such as cognitive load theory, metacognitive norms, and psychological self-regulation. The fact that most research conducted in the context of formulation remains at a descriptive level and that students are not proficient in this process highlights the need for intervention-based research to develop formulating skills.

**Methods:**

in this study, a pre-test-post-test control group quasi-experimental design was chosen to reveal the effect of the Relationships, Using Variables, Drawing Diagrams, and Simplification strategies on the ability to formulate. The participants were 52 tenth-grade students studying at an Anatolian High School in the Marmara Region of Türkiye. Two existing classes at the selected school were randomly assigned to the experimental group (*n* = 26) and the control group (*n* = 26). The experimental group received a total of 6 h of strategy-based training over 3 weeks, while the control group students continued their normal learning. The research data were obtained using a formulating test consisting of 15 formulation problems classified within the formulation process in PISA problems.

**Results:**

pre-test scores showed that both groups had similar levels of formulation ability, with no significant difference between them (*p* > 0.05). Post-test results revealed that students in the experimental group performed better than those in the control group, with a significant difference between them (Mexperimental = 8.346 vs. Mcontrol = 6.808, *t*_(50)_ = 3.161, *p* = 0.003, Cohen's *d* = 0.876). The pre-test-post-test difference for the experimental group students indicates a high level of effect (*d* = 1.081).

**Conclusion:**

the findings show that strategy-based instruction, utilizing relationships, using variables, drawing diagrams, and simplification strategies, significantly improved students' formulating skills. The strategy-based instruction designed within the framework of the Worked Examples approach has positive effects on students' cognitive load management, metacognitive awareness, and problem-solving performance. The research also contributes to the field of educational psychology both theoretically and practically.

## 1 Introduction

Education is a fundamental need for individuals, supporting their physical, mental, and psychological development (Firdaus and Wahyudin, 2017). At the same time, education plays a central role in equipping individuals with the knowledge, skills, and competencies necessary to meet the needs of contemporary society (Anil et al., 2015). For this reason, many countries view education as a strategic tool for imparting and developing various competencies in their citizens (Jailani et al., 2020). One of the most prominent outcomes of this perspective is the increasing emphasis placed on literacy skills as a fundamental educational goal (Ozgen, 2019). Accordingly, literacy is now regarded as one of the essential 21st-century competencies that education aims to impart, enabling individuals to acquire the knowledge and skills required by the modern age.

The concept of literacy has evolved beyond basic reading and writing skills to encompass higher-level abilities, such as accessing information, conducting research, thinking critically, and using knowledge to solve complex real-world problems (Jerald, 2009). In this sense, literacy now encompasses not only possessing expertise but also accessing that knowledge and using it meaningfully in various contexts. In this respect, literacy has come to be seen as one of the fundamental competencies students need to cope with the challenges of the 21st century (Drew, 2012).

Literacy is one of the key concepts in the Programme for International Student Assessment (PISA), which was conducted by the Organization for Economic Co-operation and Development (OECD). PISA focuses on how much students retain their knowledge, their potential to demonstrate their knowledge and skills, and their application of knowledge and skills in real-life situations (Organisation for Economic Co-operation and Development (OECD), 2005). In other words, PISA students live both in and out of school (Stacey and Turner, 2015). PISA aims to prepare students for social participation and lifelong learning by assessing their knowledge and skills in real-world contexts (Stacey, 2011).

PISA is one of the most effective international assessment programmes that provides important feedback for participating countries to review their education policies (İş Güzel, 2014). Countries and educators develop effective policies that can be implemented in local contexts based on the data revealed by PISA (Organisation for Economic Co-operation and Development (OECD), 2014). International assessment programmes, especially PISA, assess students' competencies at the procedural level regarding problem solving, reasoning, formulating, and using knowledge in real-life contexts. The results obtained from PISA provide the opportunity to evaluate the educational strategies of the participating countries, as well as to analyse the educational practices in successful countries and to organize and develop future educational policies accordingly. In line with the PISA evaluations, countries have made necessary adjustments to their educational policies, programmes, teacher training, and textbooks, and the development of literacy skills has become one of the most important goals of education in schools. In PISA, students' literacy competences are measured in three areas: mathematical literacy (ML), science literacy and reading skills.

ML enables individuals to understand the role of mathematics in real life (Organisation for Economic Co-operation and Development (OECD), 2013). Being literate does not only mean knowing complex mathematical formulas, analyses, linear algebra and calculations (Ojose, 2011). It can be said that a student is mathematically literate if he/she activate his/her mathematical capacities and perceptions to solve a problem or meet a need and reach a solution based on this information (Altun, 2015). One of the main objectives of mathematics teaching in schools is to develop students' ML and contribute to their ability to use and apply mathematical knowledge in solving problems in the context of real life (Sumirattana et al., 2017). Students' ML can be developed by enabling mathematical competences that form the basis of formulating, employing and interpreting-evaluation processes (Dewantara et al., 2015).

In PISA 2012 and 2022, it is observed that one of the processes that students struggle with the most in ML problems is the formulating process (Organisation for Economic Co-operation and Development (OECD), 2013; Organisation for Economic Co-Operation and Development (OECD), 2023). In parallel with these results, Edo et al. (2013) reported that secondary school students had difficulties solving problems requiring higher-level ML. These difficulties stemmed from their inability to define the structure of the problem (relationships, patterns, and order) and express it mathematically. Similarly, Ahyan and Prabawanto (2019) stated that students made mistakes in PISA mathematics problems, especially in transformation and operation skills, due to a lack of formulating skills. Muhaimin et al. (2024), in a study examining studies involving ML and mathematical problems, classified mathematical literacy problems as three basic components and revealed that one of them is difficulties in formulating mathematical situations. Sa'diyah et al. (2024) stated that students aged 14–16 were inadequate in understanding the problem situation, identifying variables, and developing solution strategies in context-based mathematical problems, and therefore had difficulties formulating solutions. Dewantara et al. (2015) and Wildani (2020) demonstrated, using numerical data, that the formulation process is the stage where students make the most mistakes. Atasoy (2020) stated that students have difficulty in transforming verbal problem situations into mathematical language and emphasized that this skill should be integrated into the teaching process. Ülger et al. (2020) stated that the most frequently reported difficulties in articles focusing on ML are related to the formulating process. However, the studies on this subject are primarily descriptive in nature. When current research and PISA results are combined, students' formulating skills, which relate to their ability to translate real-life situations into mathematical structures, have not developed sufficiently.

Research shows that the formulating process is one of the complex components of ML, both cognitively and structurally. In particular, students exhibit inadequacies in transforming real-life problems into mathematical structures, analyzing problem situations, identifying relationships and variables, and constructing appropriate mathematical representations (Stacey, 2011; Edo et al., 2013). Procedural errors and inadequate mathematical thinking, modeling and strategy development skills cause these deficiencies. In this context, the systematic teaching of problem-solving (PS) strategies will support students in understanding the problem and developing mathematical expressions that are appropriate to the situation. It is thought that strategy use will contribute to overcoming the difficulties, especially in the formulation stage, by structuring the student's thinking process. Considering that most of the studies on ML in the literature remain at the descriptive level (Muhaimin et al., 2024; Ülger et al., 2020) and the insufficiency of research on formulating skills (Sa'diyah et al., 2024), integrating strategy-based teaching approaches into classroom practices may reveal more effective results for the development of these skills.

Research in the literature indicates that students experience significant difficulties in converting real-life situations into mathematical structures and representations, particularly in identifying relationships, recognizing variables, and creating appropriate representations. This situation reveals that students' formulating skills are insufficient. It is also clear that there is a lack of intervention-based studies on how to develop formulating abilities to address this situation.

This study adopts a strategy-based approach to developing students' formulating skills. Within the scope of the research, four problem-solving strategies, classified by Temel (2018) according to the formulation dimension—Finding Relationships, Using Variables, Drawing Diagrams, and Simplification—were examined within a holistic framework to investigate their effects on developing students' formulation skills. Accordingly, this study presents an assessment of the systematic integration of selected problem-solving strategies into teaching to support the development of high school students' formulation skills.

### 1.1 The conceptual framework for formulating

**Figure 1:**
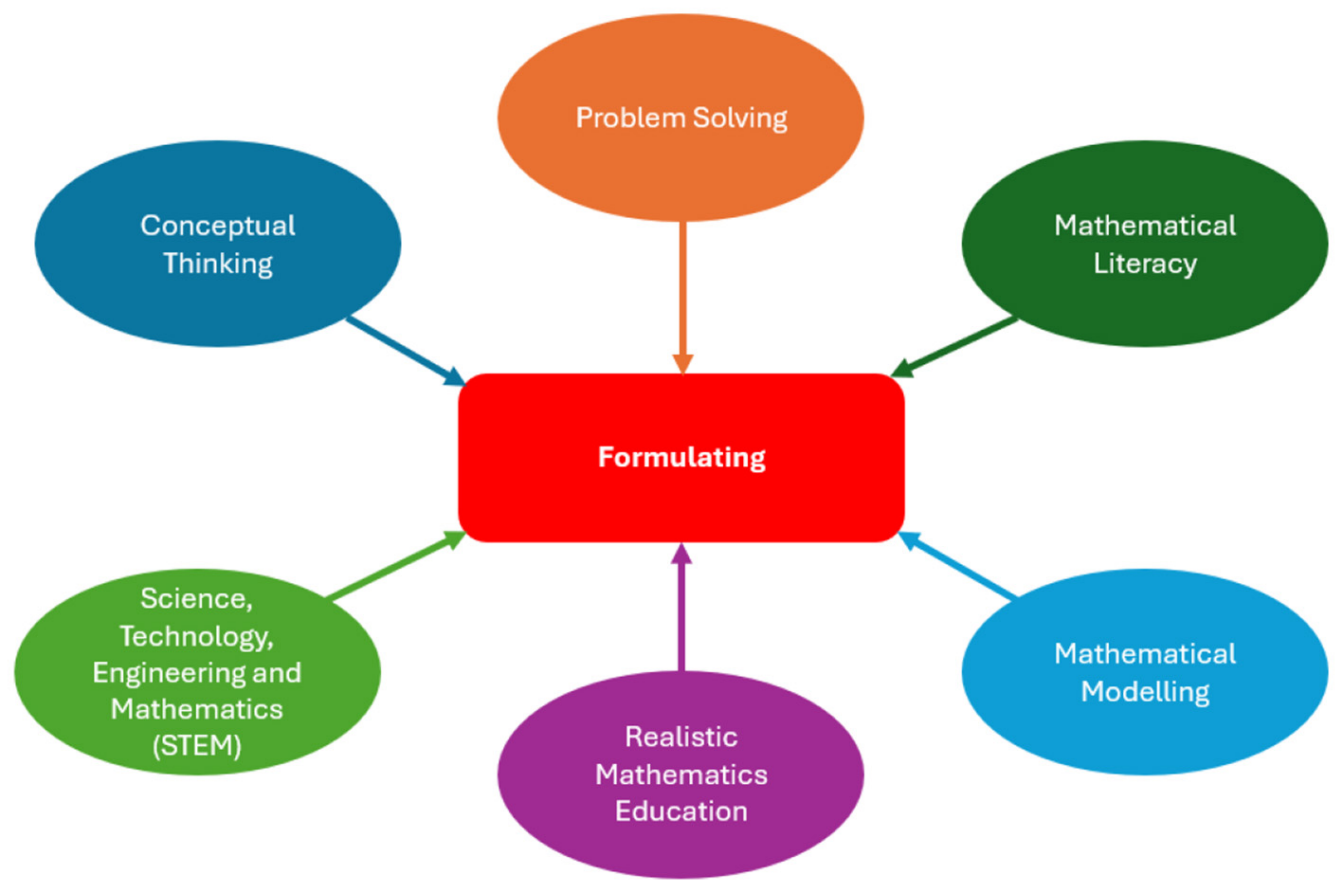
The relation of formulating to mathematical concepts.

The ability to formulate is a fundamental component of mathematics education (Luna and Díaz, 2023). Organisation for Economic Co-Operation and Development (OECD) (2023, p. 81) explains the formulating of mathematical situations as ‘mathematically literate students can recognize or identify the mathematical concepts and ideas underlying problems encountered in the real world and then provide a mathematical structure to the problems. Formulating skills include making mathematical sense of situations given in the context of the real world (Organisation for Economic Co-Operation and Development (OECD), 2023), reaching generalizations in the process of transforming mathematical expressions into a systematic structure (Tall, 2013), interpreting abstract concepts into real world contexts (Blum and Niss, 1991), and transforming the applications of mathematical abstractions in real life into problem situations. When the process of making sense of the basic concepts in mathematics education and the topics in the focus of mathematics education in recent years such as problem solving (PS), mathematical literacy (ML), mathematical modeling, realistic mathematics education (RME), Science, Technology, Engineering and Mathematics (STEM) education and computational thinking are examined, it will be seen that these concepts contain a formulating process (see Figure 1).

Mathematical modeling, as a fundamental concept in mathematics education, involves expressing real-life situations in mathematical terms (Dundar et al., 2012). This process consists of several stages, including determining the real-life problem, creating an appropriate mathematical representation, finding a mathematical solution, interpreting the solution, and evaluating the results obtained (Stillman et al., 2007). When the models that emerged in the context of mathematical modeling in the literature are examined, it is seen that processes such as formulating real-life situations (Berry and Houston, 1995; Blomhøj and Jensen, 2007; Mason, 1988; Voskoglou, 2006) and formulating the model (Berry and Houston, 1995) are included in the models. These designs show that formulating is an integral part of mathematical modeling.

Formulating plays a critical role not only in the mathematical modeling process but also in transforming real-life problems into mathematical contexts and their solutions. In this context, it is observed that formulating is involved in both horizontal and vertical mathematisation processes in RME (Artut and Bal, 2016; Demirdöǧen and Kaçar, 2010; Zulkardi, 2000). In the horizontal mathematisation process, concrete problems are transformed into mathematical concepts (Treffers, 2012) and a transition from real-life contexts to the world of mathematical symbols (Doorman and Gravemeijer, 2009). In other words, in this process, real-life problems are given a mathematical structure. (Van den Heuvel-Panhuizen and Drijvers (2020) define horizontal mathematisation as the process of transition from daily life to symbolic representations. According to Sancu and Şahinkaya (2023), the equivalent of formulating within the framework of ML in RME is the organization and mathematical expression of data in the process of horizontal mathematisation. Vertical mathematisation, conversely, involves reaching generalizations by transforming mathematical expressions into systematic and abstract structures in the focus of formulating (Tall, 2013). Treffers (2012) defines vertical mathematisation as the process of transforming concrete problems into abstract mathematical structures. In this context, Ayvali (2013) shows the process of formulating a relation and creating a mathematical model as examples of vertical mathematisation.

In STEM education, which is another critical issue in mathematics education, formulating the problems that can be answered within the framework of scientific research and formulating the problems that can be solved through engineering design, followed by construction and evaluation, are carried out through science, technology, engineering and mathematics disciplines (Kennedy and Odell, 2014). Advocates of K-12 STEM education argue that implementing STEM education in the context of real-world problems can make teachers and students more interested and motivated in STEM subjects (Honey et al., 2014). The realization of successful STEM learning depends on students' generation, conceptualization and application of ideas for real-world problems (National Research Council [NRC], 2011; Kelley and Knowles, 2016). Based on the conceptualisations and underlying explanations of STEM education, STEM teaching requires integrating a set of conceptual and procedural content within the framework of STEM skills to solve interdisciplinary problems or implement ideas in real-life contexts (Martín-Páez et al., 2019). In this context, formulation is employed in STEM education to transfer real-life problems into the mathematical world, integrate conceptual and procedural content related to real-life problems, or derive generalizations in solving problems based on science, technology, engineering, and mathematics content.

When the term computational thinking is analyzed, it is defined as the expression of the thinking processes involved in formulating and solving problems effectively by a computer (Wing, 2006). Ng and Cui (2021) demonstrated the necessity of formulating skills in their study, which was conducted with fifth and sixth-grade students within the framework of computational thinking-based mathematical problem-solving activities. As can be seen from the definition of computational thinking and the studies on computational thinking, formulating is one of the critical concepts for computational thinking. Each algorithmic thinking process begins with a process of understanding and formulation that results in abstracting real-world problems (Lehmann, 2024). Lehmann (2024), while examining the effect of the mathematical modeling process on students' algorithmic thinking, emphasizes the necessity of formulation in these processes.

PS has been a significant research area and theme in mathematics education for the past four decades (Santos-Trigo, 2024). Mathematicians and mathematics educators agree that PS plays a central role in both mathematical discovery and mathematics teaching (Fried, 2014). Focusing on recent research and trends in PS in mathematics, it is argued that progress in mathematical practice revolves around two intertwined activities: formulating mathematical problems and approaching and solving problems (Santos-Trigo, 2024). PS forms the basis of mathematical thinking and discoveries, while formulating is the initial stage of this process. When Polya's (1945) ideas are analyzed, it will be seen that formulating and solving problems are the activities at the center of the development of the mathematics discipline. (Polya 1945) analyzed the PS process in four stages: understanding the problem, making a plan, implementing the plan and evaluating the results. Formulating is directly related to the first stage of this process, “understanding the problem”. Formulating is the process of transforming real-life problems into a mathematical structure. Levenberg and Shaham (2014) and Morgan (1996) refer to students' formulating skills in solving verbal problems. The formulation process can be seen as the first step in problem-solving and involves understanding problems and expressing them in mathematical terms. Mathematical formulation skills contribute to the development of the mathematical discipline by enabling the understanding of complex problems and the creation of conceptual structures for their solution.

The formulating process acts as a bridge between the real world and the mathematical world. In this context, the models of ML, realistic mathematics education and mathematical modeling processes model the processes between the real world and the mathematical world. When the models for ML are examined, the ability to formulate appears as a key concept in the transition from real life to the mathematical world (Organisation for Economic Co-operation and Development (OECD), 2013; Organisation for Economic Co-Operation and Development (OECD), 2023). In the PISA report by Organisation for Economic Co-Operation and Development (OECD) (2023, p. 40), ML is defined as “students” capacity to reason mathematically and formulate, employ and interpret mathematics to solve problems in various real-world contexts. The formulation process plays a critical role in the basis of ML.

In summary, formulating skills, which are involved in critical processes in mathematics education such as mathematical modeling, RME, and STEM education, play a significant role in developing individuals' mathematical thinking and PS competencies. Research shows that the formulation process is not only a mathematical concept but also closely related to an individual's ML and PS skills. Formulating, which is used in transforming real-life problems into mathematical structures, supports individuals in producing symbolic representations and developing effective PS ways by using these representations. Therefore, it can be said that there is a structural and functional link between formulating and PS skills. Both horizontal and vertical mathematisation processes support this link and contribute to the strengthening of the individual's ML level. Moreover, formulating problems is a critical component of ML, closely related to practical PS skills. PS strategies or problem-based instruction focusing on developing students' formulating skills can contribute significantly to ML and PS skills.

### 1.2 The cognitive and psychological dimension of formulating skills

Mathematical formulation ability, while being a mathematical process, is directly related to cognitive load management (Sweller, 1988), problem representation formation (Jonassen, 2011), and levels of metacognitive awareness (Veenman et al., 2006). Research reports indicate that the effective teaching of mathematical formulation requires the integrated consideration of cognitive load theory (CLT), metacognition, and different types of reasoning (Asmara et al., 2024; Phan et al., 2017; Tak et al., 2022). The difficulties students encounter when converting real-life situations into mathematical structures are closely linked to the cognitive strategies employed in the problem-solving process (Schoenfeld, 1985) and self-regulation mechanisms (Zimmerman, 2002).

When examining the problem situations students encounter today, it is expected that students will create mathematical structures appropriate to the situation and reveal the relationships between these structures, rather than merely solving the given situations mechanically. This is because the instructions consist of situations that require unclear understanding, the visuals require verbal explanations, and the tasks are presented with increasing complexity. In this context, to formulate solutions for increasing complexity in problem-solving, situations must be related to mathematical concepts, generalized, and interpreted. Studies conducted within the context of cognitive load theory show that such situations develop students' formulation skills (Asmara et al., 2024; Phan et al., 2017). These situations demonstrate that the development of formulation skills is related not only to mathematical situations but also to how cognitive load regulates the environment.

Another mental process that contributes to the completion of formulation and the ability to formulate correctly is metacognitive awareness. Cognitive processes that regulate the formulation process, such as planning, monitoring, and evaluation, help students decide which representation is appropriate and test the accuracy of the model they have created (William and Maat, 2020). In this context, it can be said that the ability to formulate is closely related to cognitive skills, including mathematical skills, as well as psychological self-regulation tools, which are metacognitive activities (Zimmerman, 2002).

In the design of instructional applications, considering cognitive load theory and integrating metacognitive norms into this process can significantly improve students' formulation skills. Under these circumstances, it is necessary to eliminate tasks that unnecessarily strain students' working memory and distract them, focusing instead on the actual formulation processes (Richland et al., 2017). Therefore, when designing instruction, it is recommended to incorporate practices that balance cognitive load, such as gradual complexity, step-by-step representations, and comparison-based activities (Phan et al., 2017; Tak et al., 2022; Hartelt and Martens, 2024). In terms of teaching strategies, applications such as metacognitive education, worked examples, context-based problem-solving, and collaborative metacognition stand out as practical approaches for developing formulation skills (Dhlamini, 2016; Hartelt and Martens, 2024; Tachie, 2019). In terms of assessing formulaic reasoning skills, expert-rated assessments using structural equation models (Tak et al., 2022) reveal significant gaps in this field, including a lack of causal evidence, limitations of measurement tools, and a scarcity of in-depth research. Therefore, it is considered important that studies aimed at developing formulation skills focus on both experimental and classroom-scale applications to address this gap in the field.

### 1.3 Purpose and significance of the study

ML is seen as a fundamental competence that includes the ability of individuals to mathematically understand, formulate, solve and interpret the results of the problems they encounter in daily life (Organisation for Economic Co-operation and Development (OECD), 2013). However, research indicates that the formulation process, one of the processes within these competency components, is the most challenging stage for students (Ahyan and Prabawanto, 2019; Edo et al., 2013; Muhaimin et al., 2024; Ülger et al., 2020; Sa'diyah et al., 2024; Wildani, 2020). In PISA 2012 and 2022, it is evident that formulating problems is among the most challenging processes for students (Organisation for Economic Co-operation and Development (OECD), 2013; Organisation for Economic Co-Operation and Development (OECD), 2023). International research indicates that similar difficulties are observed not only in Türkiye but also in different education systems (Blum and Ferri, 2009; Kaiser and Stender, 2013) and that formulating these difficulties is considered a fundamental cognitive process, particularly in the context of STEM education (Honey et al., 2014) and computational thinking (Wing, 2006; Ng and Cui, 2021).

The formulating process is multifaceted and requires complex cognitive skills, such as converting real-life situations into mathematical structures, identifying variables, and developing appropriate solution strategies in the context of problem-solving. Therefore, students must have well-developed formulating skills to succeed with higher-level mathematical problems. However, the literature emphasizes that students often experience difficulties with such issues. (Edo et al. 2013) showed that students' failure on advanced mathematical modeling problems is due to their inability to recognize the problem structure and frame situations in a mathematical context. These findings are supported by studies reporting that students aged 14–16 experience difficulties in understanding the context of a problem, identifying variables in context-based issues, and developing strategies (Sa'diyah et al., 2024). These situations demonstrate that the root of students' difficulties lies in their lack of formulating skills. However, the limited number of studies on the formulating process in the literature, combined with the fact that most existing studies remain at a descriptive level (Muhaimin et al., 2024; Ülger et al., 2020), clearly demonstrates the need for a more in-depth and application-oriented examination of this process.

In his study, Temel (2018) classified PS strategies according to mathematical process skills and determined that “Finding Relationships”, “Using Variables”, “Drawing Diagrams”, and “Simplification” strategies can contribute to the formulating process. In this context, teaching PS strategies has the potential to eliminate the difficulties experienced in the formulation process. Systematically teaching these strategies to students can improve their ability to transform real-life problems into mathematical language. However, there are insufficient, systematically tested studies that do not support this hypothesis.

The primary purpose of this study is to examine the effects of PS strategies on students' development of formulation skills. The effects of “Finding Relationships”, “Using Variables”, “Drawing Diagrams”, and “Simplification” strategies on formulating abilities were examined in this context. Within the scope of the research, the effect of strategy-based instruction was analyzed by keeping the instructional content constant. Unlike previous studies, this study examines the selected problem-solving strategies independently. It adopts an integrated approach by simultaneously focusing on four strategies to develop formulating skills.

It is foreseen that the research will contribute to both the ML literature and teaching practices and develop a concrete and applicable model for solving the existing problems related to the formulating process. Another reason for conducting this research is to fill the aforementioned gap by examining the effect of strategy-based teaching on students' formulation skills within an experimental design framework. Furthermore, the findings are expected to contribute not only to the field of mathematics education but also to the literature on learning psychology and cognitive sciences by offering original insights into the cognitive and metacognitive mechanisms that support students' problem-solving processes.

Moreover, when Türkiye's ML performance in PISA 2003–2022 is analyzed, it is seen that Türkiye's ML score is below the average of OECD member countries (Ministry of National Education (MoNE), 2023). Although there has been a significant increase in this process, it can be said that this increase is insufficient. The study is also noteworthy in that it offers evidence-based recommendations for countries such as Türkiye that are striving to develop education policies to improve their PISA performance.

### 1.4 Research problem

How does strategy-based instruction (Finding Relationships, Using Variables, Drawing Diagrams and Simplification) affect students' success in the formulating process?

#### 1.4.1 Sub-problems

1. What is the current level of students' formulating skills (Is there a significant difference between the pretests of the experimental and control group students)?
2. Does “Finding Relationships”, “Using Variables”, “Drawing Diagrams” and “Simplification” strategy training significantly affect students' formulation skills (Is there a significant difference between the post-tests of the experimental group and control group students)?

## 2 Method

In this study, the effects of the “Finding Relationships”, “Using Variables”, “Drawing Diagrams”, and “Simplification” strategies on students' formulation process were examined. For this purpose, a quantitative research method was employed, allowing for the testing of theoretical approaches (Creswell, 2013). Quantitative research is based on a systematic and planned design. In this context, experimental design was adopted to reveal cause-and-effect relationships (Johnson and Christensen, 2012). Considering the limitations of educational environments where individuals cannot be randomly assigned to groups, a quasi-experimental design was used (Gopalan et al., 2020; Cohen et al., 2000). The quasi-experimental design is widely accepted as an effective method for obtaining reliable data in cases where random assignment of samples is not feasible (Marczyk et al., 2005). In fact, according to the What Works Clearinghouse (What Works Clearinghouse (WWC), 2022) standards, quasi-experimental designs rank second in terms of evidence, following authentic experimental designs. The use of quasi-experimental design enhances causal claims in educational research (Loeb et al., 2017). Within the framework of these considerations, the quasi-experimental design was found appropriate for the research.

Table 1 shows the experimental design. The two existing classes were randomly assigned by drawing lots, with one group serving as the experimental group and the other as the control group. The experimental group received training on “Finding Relationships,” “Using Variables,” “Drawing Diagrams,” and “Simplification” strategies. No intervention was made to the control group.

**Table 1:** The experimental design of the study.

**Group (*N* = 52)**	**Pre-test**	**Intervention**	**Post-test**
Experimental group (n1 = 26)	O1	X	O2
Control group (n2 = 26)	O1		O2

O1: Pre-test for formulating skills.

O2: Post-test for formulating skills.

X: Training on “Finding Relationships”, “Using Variables”, “Drawing Diagrams” and “Simplification” strategies.

### 2.1 Participants

The study was conducted with 52 tenth-grade students enrolled at an Anatolian High School in the city center of a province in Türkiye's Marmara Region. The research school was selected based on factors such as providing the necessary academic support to the researcher, willingness to participate in scientific studies, and cooperation in the implementation process. Furthermore, the fact that the vast majority of students attending secondary schools in Türkiye are educated at Anatolian high schools is another reason for choosing this school.

There are several reasons for conducting the study at the 10th-grade level. Firstly, the fact that PS skills significantly develop toward higher grade levels (Işık and Kar, 2011) provides a theoretical basis for this choice. In addition, studies on PS strategies are primarily concentrated at the secondary school level (Altun and Arslan, 2006; Arsuk and Memnun, 2020; Gür and Hangül, 2015), but they are not sufficiently addressed at the high school level. However, the fact that PISA applications target students in the 15-year-old age group makes it appropriate to conduct a study with participants at the 10th-grade level in terms of both age compatibility and measurement of formulation skills.

In the study, a quasi-experimental design with a pretest-posttest control group was applied. Accordingly, the participants were randomly assigned to the experimental and control groups based on their current class structures. Information about the demographic characteristics of the students participating in the experimental process is presented in Table 2.

**Table 2:** Demographic information of the participants.

**Group**	**Number of students (*N*)**	**Grade**	**Average age**	**Gender**			
				**Female (** ***N*** **)**	**%**	**Male (** ***N*** **)**	**%**
Experimental	26	10^th^	15.04	12	46.2	14	53.8
Control	26	10^th^	15.08	15	57.7	11	42.3
Total	52	10^th^	15.06	27	51.9	25	48.1

When Table 2 is analyzed, it is seen that the experimental group consisted of 26 tenth-grade students, 12 (46.2%) female and 14 (53.8%) male. The control group comprised 15 (57.7%) female and 11 (42.3%) male students. Of the 52 study participants, 27 (51.9%) were female and 25 (48.1%) were male. Data collection from participants and the instructional process with the experimental group were conducted in accordance with the approval of the relevant ethics committee.

In educational research, since existing class groups are used and these group sizes are fixed, the minimum detectable effect size (MDE) was calculated to assess the statistical power of the sample. For a two-sample *t*-test (two-tailed) with assumptions of α = 0.05 and power (1-β) = 0.80, the MDE was found to be approximately Cohen's *d* = 0.78, assuming *n* = 26 for each group. As the observed effect size (for post-test comparison, *d* = 0.876) exceeds this value, it can be stated that the current sample size has sufficient statistical power.

### 2.2 Data collection tool

In the study, a “Formulating Test (FT)” was used to assess the level of high school students' formulating skills and to compare their skills before and after the experimental process.

#### 2.2.1 Formulating test (FT)

The Formulating Test (FT), administered to tenth-grade students as a pre-test and post-test, comprises 15 problems related to the formulating skill area among the PISA mathematical literacy problems made available by OECD and MoNE in Turkish. This test scored correct answers as “1”, and incorrect and blank answers as “0”.

The opinions of four field experts were consulted to ensure the test's content validity. All items were confirmed to be appropriate for the purpose, content, and target student level. Then, the comprehensibility, linguistic accuracy, and applicability of the test items were evaluated using the pilot application.

Following the pilot study, the test was administered to 562 students from various high schools to assess its reliability, and the KR-20 internal consistency coefficient was calculated. As a result of the analyses, the KR-20 value of the test was determined to be 0.84, indicating high overall internal consistency. In addition, the distribution of the questions in the test according to difficulty levels was as follows: four difficult, seven medium, and four easy items. Item discrimination indices ranged from 0.31 to 0.84. These findings indicate that the FT is both a valid and reliable measurement tool.

### 2.3 Instructional design

In Temel's (2018) study, PS strategies were classified based on mathematical process skills; especially, “Finding Relationships,” “Using Variables,” “Drawing Diagrams,” and “Simplification” strategies stand out in the formulating process. Considering this situation, it is thought that training on these strategies can improve students' formulating skills.

Accordingly, within the scope of the permission granted by MoNE, 26 tenth-grade students in the experimental group were trained on the “Finding Relationships”, “Using Variables”, “Drawing Diagrams”, and “Simplification” strategies. The training consisted of problem-solving and strategy-oriented applications lasting six course hours for 3 weeks, starting after the pre-test application. Following the training, post-test applications (one course hour) were conducted, and the implementation process was completed within 5 weeks.

Before the training on strategies, similar applications in the literature were examined (Altun and Arslan, 2006; Altun et al., 2004; Bülbül and Taş, 2023; Ulu et al., 2016; Verschaffel et al., 1999; Yazgan and Bintaş, 2005). In most of these studies, problems that can be solved with specific strategies were presented to students and training was carried out by discussing these problems.

Problem pools specific to each PS strategy were created for the training process. In the preparation of these pools, the studies by Posamentier and Krulik (2008) and Krulik and Rudnick (1989) were utilized. Four problems were selected for each strategy, and lesson plans were prepared to teach the relevant strategy in line with these problems. The lesson plans were based on the teaching plans developed by Taşpinar (2011), Dhlamini's (2016) Worked Examples approach and the four-stage process defined by Polya in the PS process (understanding the problem, creating a solution plan, implementing the plan and evaluating the solution). In instructional design processes, attention has been paid to the inclusion of processes that balance cognitive load, such as activities based on comparison, where different representations are used to solve problems, revealing multiple solutions to a problem and comparing solutions obtained from different formulations. In this context, Dhlamini's (2016) Worked Examples approach, based on Cognitive Load Theory (CLT), has also been taken into consideration in the design of training. This approach aims to present clear and step-by-step solutions in the learning process, focusing on the stages of understanding the problem, transforming it into a mathematical representation, and formulating it. Within this framework, the aim is for students to determine what is required in problems, establish relationships between what is given and what is required, analyse these relationships, and express them using appropriate equations, shapes, mathematical expressions, or formulas. Thus, the designed strategy in education aims to deepen students' conceptual understanding by balancing their cognitive load and increasing their formulation competencies through the development of problem-solving skills.

Each strategy training was planned to be implemented during one lesson hour. The clarity and comprehensibility of the instructions for the draft lesson plans, as well as the suitability of the problem content to the students' level, were evaluated based on the opinions of two professors and one assistant professor. The necessary arrangements were made and finalized. Within the framework of the lesson plans, problems related to each strategy were discussed during the implementation process, and solutions suitable for the respective strategies were explored in the classroom environment.

**Figure 2:**
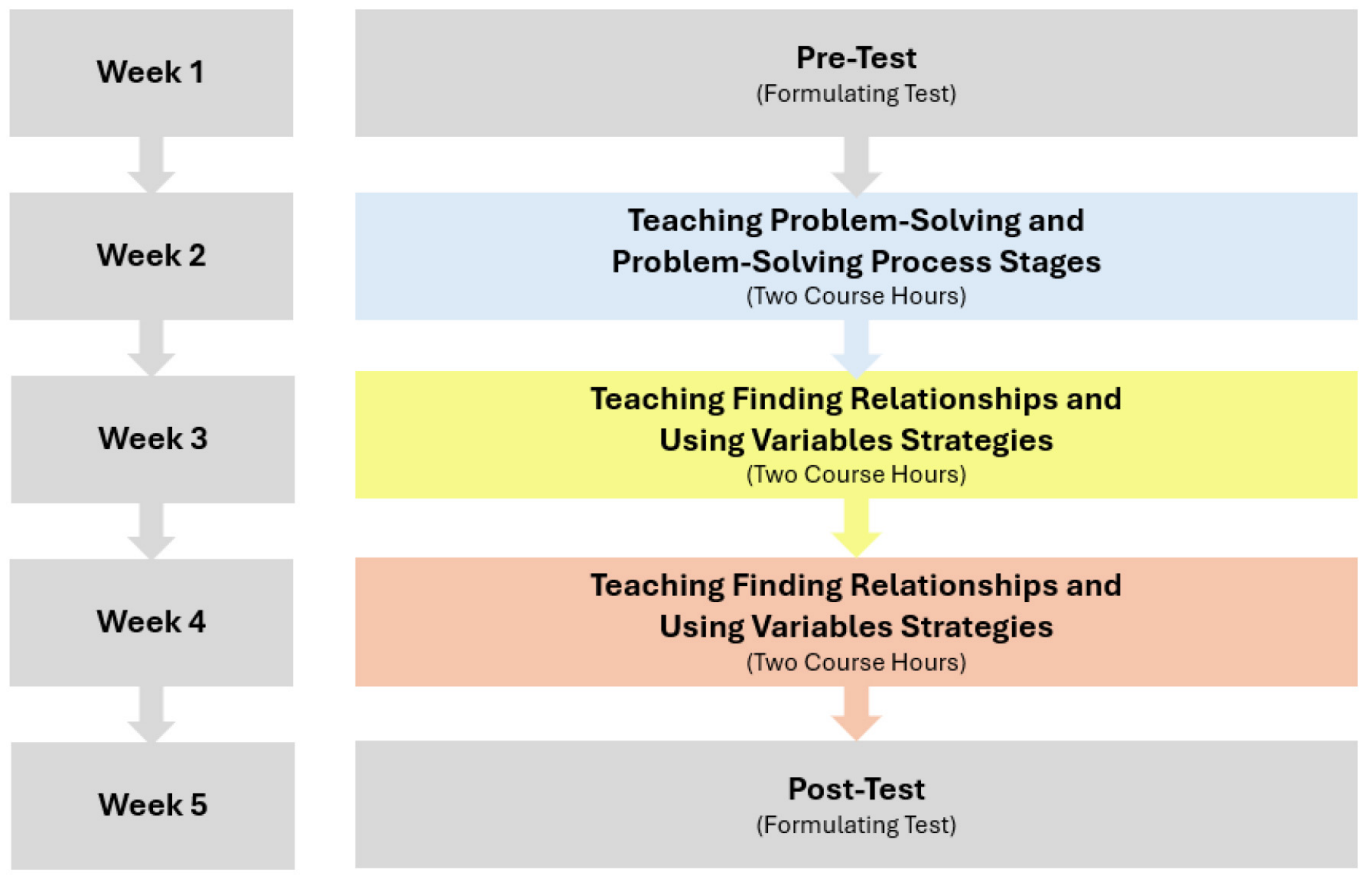
The instructional design.

Figure 2 shows the weekly distribution of strategy trainings, course hours, and the teaching design process.

In Table 3, the themes for teaching strategies and the corresponding targeted learning outcomes are presented in detail.

**Table 3:** Weekly distribution of strategy trainings and learning outcomes.

**Week**	**Theme**	**Learning outcome**
1. Week (2 Course hours)	Problem-solving and problem-solving process stages	• To understand the importance of problem-solving • To be able to use problem-solving steps while solving problems • To be able to benefit from the stages of the problem-solving process
2. Week (2 course hours)	Finding relationships and using variables	• Using the stages of the problem-solving process • Establishing relationships between what is given and what is desired, and analyzing these relationships • To be able to realize problem-solving strategies
3. Week (2 Course Hours)	Drawing diagrams and simplification	• To be able to create equations, diagrams, mathematical expressions or formulas appropriate to the relationships in the problem • To be able to make a judgment for the selection of the appropriate strategy for solving problems • To be able to decide on the use of the appropriate strategy for solving problems • To be able to utilize the relevant strategy in solving problems • Explain why the chosen strategy is used • To be able to use different strategies in cases where the solution cannot be reached with the selected strategy • To be able to infer that more than one strategy can be used in solving problems • Reasoning whether the solved problem can be solved with different strategies • Solve real-life problems

### 2.4 Data analysis

In the study in which the effects of “Finding Relationships”, “Using Variables”, “Drawing Diagrams” and “Simplification” strategies on formulating skills were experimentally examined, the data obtained by applying FT to the students were transferred to the Statistical Package for Social Sciences for Personal Computers (SPSS) programme and various statistical measurements and analyses were carried out within the framework of the research problems.

FT scores were evaluated based on Item Response Theory and Bloom's Cognitive Domain Theory (Anderson and Krathwohl, 2001; Lok et al., 2016). A criterion-based approach was adopted in the evaluation, and achievement levels were categorized according to objective criteria. Accordingly:

- High level (11–15 points): competence in solving complex problems, analysis, synthesis and evaluation skills,
- Intermediate level (6–10 points): competence in solving, comprehending and applying easy and intermediate problems,
- Low level (0–5 points): shows a lack of basic knowledge and understanding, an inability to formulate.

Before applying a parametric or non-parametric test on the data obtained, it is necessary to evaluate whether the normality assumption is met (Pallant, 2020; Field, 2024). Testing assumptions of normality, identifying potential violations, and selecting appropriate alternative analysis methods are critical to enhancing the validity of results (Can, 2014). Various methods can be used to examine normality assumptions in this direction, including descriptive statistics (such as skewness and kurtosis values), Q-Q plots, and normality tests (Field, 2024; Ghasemi and Zahediasl, 2012). In this study, the normality assumption was evaluated using multiple methods. During the examination process, attention was paid to the skewness and kurtosis values, which were within the range of −1 to +1 (Tabachnick and Fidell, 2019). A *p*-value greater than 0.05 was used as the criterion in the Shapiro-Wilk test (Razali and Wah, 2011), the strongest normality test recommended, especially for small sample sizes.

In educational research, the *t*-test is recommended for comparing the achievement scores of two groups (Fraenkel et al., 2011). Accordingly, the *t*-test for unrelated samples, one of the parametric analysis methods, was used to determine the significant differences between the pre-test and post-test scores of the experimental and control groups. A paired *t*-test was used to compare the pre-test and post-test scores of both groups. Normality assumption was evaluated according to the criteria that skewness and kurtosis values were in the range of −1 to +1 and the *p* value of the Shapiro-Wilk test was greater than 0.05. Variance homogeneity was checked with Levene's test, and *p* > 0.05 was found (Tabachnick and Fidell, 2019).

In interpreting *t*-test results, Lakens (2013) emphasizes that the effect size (Cohen's *d*) must be reported. In addition, according to the What Works Clearinghouse (What Works Clearinghouse (WWC), 2022) standards, the size of the difference between pre-tests in experimental research is considered when determining the post-test analysis method. Accordingly, if the effect size is below 0.05, applying a direct *t*-test in post-test comparison is considered appropriate. If the effect size is between 0.05 and 0.25, statistical adjustments such as ANCOVA are needed in post-test analysis (What Works Clearinghouse (WWC), 2022).

In this study, since the effect size in the pre-tests of the experimental and control groups was calculated to be below 0.05, a *t*-test analysis for unrelated samples was preferred for comparing post-test results.

### 2.5 Validity and reliability

Validity and reliability are the primary concepts that ensure the accuracy, meaningfulness, and reliability of findings in educational experimental research. Validity refers to the extent to which a study or research tool measures what it wants to measure accurately or appropriately. In contrast, reliability refers to the ability to obtain the same results repeatedly by conducting the study multiple times (Johnson and Christensen, 2012). Both concepts are widely accepted as the basic criteria for evaluating research in educational sciences (Fendler, 2016). In this context, the criteria and threats to validity and reliability emphasized in the studies of Creswell (2013), Fraenkel et al. (2011) and Cook and Campbell (1979) and how they were overcome for the study are discussed below:

To prevent threats related to subject characteristics, a form containing various demographic information, such as age, gender, and grade level, was prepared for the participants. It was ensured that the experimental and control groups had similar backgrounds. To mitigate the risk of attrition, a voluntary participation process was implemented, and no subjects were lost during the study. In order to reduce the Hawthorne effect, which has the risk of changing the behavior of the participants due to the knowledge that they are being observed, the training sessions were conducted within the scope of the course, and the participants were not informed that the study was an experiment or which group they were in.

The interventions were carried out simultaneously in the experimental and control groups to prevent time-related threats. In addition, the fact that the study was conducted with 10th-grade students ensured that the process was completed within a reasonable time frame, and comparisons between groups minimized threats related to maturation. Since the achievement levels of the groups were close to each other before the intervention, and the control group was also included in the study, the regression effect (regression to the mean) was prevented.

To minimize the threat of selection bias, the pre-test results revealed the initial equivalence of the groups, and it was determined that the experimental and control groups were equivalent. The applications were conducted in classrooms with similar physical conditions to reduce the threat of external validity, and the effect of environmental factors was minimized.

The lesson plans, prepared in advance, were used to implement the training process, ensuring consistency in the research. To increase the accuracy of the data, an independent expert evaluation, in addition to that of the researcher, was also applied during the coding and evaluation of the student responses; thus, data reliability was ensured by employing a double control method.

Expert opinions were consulted during the development of the measurement tool to ensure content validity, and the internal consistency of the measurement tool was evaluated by calculating the KR-20 reliability coefficient.

## 3 Results

This section presents the study's results to determine the effect of “Finding Relationships”, “Using Variables”, “Drawing Diagrams” and “Simplification” strategies on students' formulating skills. Within the scope of the study, pre-test and post-test data were collected using the FT applied to the experimental and control groups. The results obtained were analyzed and presented systematically within the framework of the research problems. In presenting the results, the ways in which these strategies contribute to the development and differences in students' formulation processes are discussed.

### 3.1 Results about the current levels of students' formulating skills

To determine the significant difference between the pre-tests of the experimental and control groups, the normality of the data was examined first. The skewness and kurtosis values of the experimental and control groups were 0.23 and −0.22, respectively, and −0.34 and 0.69, respectively, indicating that the data were normally distributed. In addition, the significance values obtained in the Shapiro-Wilk normality test results are greater than 0.05, which supports this situation. Within the framework of these findings, it was accepted that the data groups were normally distributed. *T*-test for unrelated samples, which is one of the parametric analysis methods, was used to determine whether there was a significant difference according to the FT pre-test scores of the tenth-grade students in the experimental and control groups before the “Finding Relationships”, “Using Variables”, “Drawing Diagrams” and “Simplification” strategies training. The *t*-test analysis results for unrelated samples are presented in Table 4.

**Table 4:** T-test results for pre-tests.

**Group**	***N***	**M**	**SD**	***df***	***t***	***p***	**%95 Conference interval (ci) lower-upper**		**Cohen *d***
Experimental	26	6.423	1.528	50	−0.169	0.867^*^	−0.992	−0.838	0.047
Control	26	6.500	1.750						

^*^p > 0.05.

When Table 4 is examined, it is seen that there is no significant difference between the FT pre-test scores of the experimental and control group students (*t*_(50)_ = −0.169, *p* = 0.867, mean difference = −0.077, 95% CI [−0.992, 0.838]). According to What Works Clearinghouse (WWC) (2022) standards, the analyses to be performed for the post-test in an experimental study are determined based on the effect size. Accordingly, Cohen's *d* effect size was calculated in this study. Effect size can be defined as an index that measures the magnitude of the relationship between variables (Hedges, 2008). In other words, effect size is a statistical indicator that reflects the deviation of the results obtained from the sample from the expected results under the null hypothesis (Cohen, 1994). Today, many educational journals expect researchers to report effect size measurements in addition to *t*- or *F*-tests (Huberty, 2002). In this context, effect size is a crucial concept in educational research (Richardson, 2011). Therefore, the effect size was calculated and interpreted in this study. Within the framework of the calculated effect size, a *t*-test analysis was performed on unrelated samples to determine the significant difference between the post-tests of the experimental and control groups.

Considering the pre-test scores, it was determined that the average score for the experimental group was 6.423, and the average score for the control group was 6.500. This finding indicates that the formulation levels of most students in both groups were at a medium level. According to the FT pre-test scores, it was determined that seven students in both the experimental and control groups had low-level formulating skills, and 19 students had medium-level formulating skills.

### 3.2 Results on the effects of “finding relationships”, “using variables”, “drawing diagrams” and “simplifying” strategy training on students' formulating skills

After the “Finding Relationship”, “Using Variables”, “Drawing Diagrams” and “Simplification” strategies training with the experimental group, FT was applied to the students in the experimental and control groups as a post-test to determine whether there was an effect on the students' formulation skills. Normality assumptions were examined to determine the difference between the groups. The calculation of the skewness values for the experimental and control groups as 0.001 and −0.213, respectively, and the kurtosis values as −0.928 and 0.990, respectively, along with the determination of significance values greater than 0.05 within the scope of the normality test results, indicate that the data groups exhibit a normal distribution. In this framework, a *t*-test analysis for unrelated samples was applied to determine whether there was a significant difference between the FT scores of the experimental and control groups, as measured by the post-tests. The results of the analysis are given in Table 5.

**Table 5:** *T*-test results for post-tests.

**Group**	***N***	**M**	**SD**	***df***	***t***	***p***	**95% CI lower-upper**		**Cohen *d***
Experimental	26	8.346	1.999	50	3.161	0.003^*^	0.561	2.516	0.876
Control	26	6.808	1.470						

^*^p < 0.01.

When Table 5 is examined, as a result of the *t*-test analysis performed according to the FT post-test scores of the experimental and control groups, a statistically significant difference was found between the experimental and control groups (*t*_(50)_ = 3.161, *p* = 0.003, Cohen's *d* = 0.876, 95% CI [0.561, 2.516]). (Hattie 2008) emphasizes that in educational research, an effect size of 0.40 indicates an average effect, and an effect size greater than 0.60 indicates a pedagogically significant difference. According to this effect size criterion, calculated using Cohen's (1988) effect size classification, this effect is considered “large”. Within the framework of these findings, it can be said that training in “Finding Relationships”, “Using Variables”, “Drawing Diagrams”, and “Simplification” strategies strongly affects students' formulation skills.

The mean FT score of the experimental group (*M* = 8.346) was significantly higher than that of the control group (*M* = 6.808). In line with these findings, it can be said that most students in both groups have medium-level formulating skills. According to the post-test results, two students in the experimental group showed a low level, 19 students showed a medium level, and five students showed a high level of formulating skills. Four students in the control group had low-level formulating skills, and 22 had medium-level formulating skills. Compared to the pre-test results, it was determined that five students in the experimental group progressed from a medium to a high level, and the number of students with low formulation skills decreased from seven to two.

These results show that training on the “Finding Relationships,” “Using Variables,” “Drawing Diagrams,” and “Simplification” strategies positively improved the students' ability to formulate levels. In addition, the data revealed that the strategy training increased formulating skills in a statistically significant and pedagogically transformative way.

The effects of the training for “Finding Relationships”, “Using Variables”, “Drawing Diagrams” and “Simplification” strategies on the students' formulating skills were examined by comparing the groups. In this context, the difference between the pre-test and post-test scores of the experimental group was analyzed using a paired *t*-test. The findings related to the analysis are presented in Table 6.

**Table 6:** *T*-test results comparing the pre-test and post-test scores of the experimental group.

**Experimental group**	***M***	**SD**	***df***	***t***	***p***	**95% CI**		**Cohen *d***
						**Lower**	**Upper**	
Pre-test	6.423	1.528	25	−7.088	0.000^*^	−2.482	−1.364	1.081
Post-test	8.346	1.999						

^*^p < 0.001.

According to the findings in Table 6, it was concluded that the post-test scores of the experimental group (*M* = 8.346, SD = 1.998) were significantly higher than the pretest scores (M = 6.423, SD = 1.527) (*t*_(25)_ = −7.088, *p* < 0.001, Cohen's *d* = 1.081, 95% CI [−2.482, −1.364]). These findings support that strategy training has a significant effect on formulation skills.

To eliminate the threat of internal validity, a pretest-posttest analysis was also conducted for the control group (Shadish et al., 2002). Accordingly, as in the experimental group, the difference between the pre-test and post-test scores of the control group was analyzed using the paired *t*-test. The findings obtained are presented in Table 7.

**Table 7:** *T*-test results comparing the pre-test and post-test scores of the control group.

**Control group**	**M**	**SD**	***df***	***t***	***p***	**95% CI**		**Cohen *d***
						**Lower**	**Upper**	
Pre-test	6.500	1.749	25	−1.690	0.103^*^	−0.682	0.067	0.190
Post-test	6.807	1.470						

^*^p > 0.05.

Within the framework of the findings in Table 7, it was determined that there was no significant difference between the pre-test and post-test scores of the control group (*t*_(25)_ = −1.690, *p* = 0.103, Cohen's *d* = 0.190, 95% CI [−0.682, 0.067). Although the post-test mean score of the control group (*M* = 6.807) increased compared to the pre-test mean score (*M* = 6.500), it can be said that this increase was insignificant when the effect size is considered. This minimal change in the control group suggests that the effect of the training provided to the experimental group is statistically significant, supporting the success of this intervention.

## 4 Discussion and conclusion

This study aims to examine the effects of the strategies of “Finding Relationships”, “Using Variables”, “Drawing Diagrams” and “Simplification” on the formulation process. The effects of the strategies of “Finding Relationships”, “Using Variables”, “Drawing Diagrams” and “Simplification” on the formulating skills of high school students were examined. In the process of adopting a quasi-experimental approach, training was conducted with the tenth-grade students in the experimental group on the mentioned strategies, and the effects of these strategies on the students' formulation process were examined.

Today, mathematics is not only a subject that needs to be taught, but also a fundamental discipline required for individuals to be active stakeholders in a contemporary society and contribute to sustainable development (National Council of Teachers of Mathematics (NCTM), 1989). Beyond being a discipline that only includes numbers and operations, mathematics is a tool that develops critical skills such as mathematical thinking, problem-solving, critical thinking, formulating, and reasoning (Maass et al., 2019). Therefore, contemporary mathematics curricula should structure mathematical knowledge to develop students' reasoning, critical thinking, and the ability to formulate and solve problems using mathematics (Leung et al., 2014; Tesfamicael and Enge, 2024).

Correct problem formulation is essential for an effective PS process and advanced ML. An educational process based on PS strategies can significantly improve students' ML and PS skills (Temel, 2018). The study's findings support this situation; it shows that strategy-based education increases students' PS skills and capacity to transform real-world problems into meaningful mathematical structures. This provides an opportunity for mathematics to gain a meaning that touches life beyond abstract concepts.

In light of these evaluations, one important way to increase students' ML achievement is to develop their formulation skills. The findings support the view that the PS process is more effective in developing students' ML competence compared to traditional teaching plans, as noted by Sumirattana et al. (2017). The findings obtained within the scope of the study showed that the strategies of “Finding Relationships”, “Using Variables”, “Drawing Diagrams” and “Simplification” have a strong positive effect on students' formulating skills. In this context, students with low formulating levels can be identified, and strategy-based training can be provided to them to develop their formulating skills systematically. In this way, students' capacity to transform real-life problems into mathematical models more effectively can be increased, and their mathematical thinking processes can be deepened. Therefore, such training can be widespread, especially in areas that require mathematical modeling or scientific process skills.

The formulation process transfers a problem or situation in the context of real life to the mathematical world. In other words, formulating is connecting real life with the mathematical world, by determining the contribution of “Finding Relationships”, “Using Variables”, “Drawing Diagrams” and “Simplification” strategies to the formulating process, the design of trainings to be carried out for the development of ML process skills, the selection of problems to be used in lessons and ML trainings to be carried out for teachers and teacher candidates can be re-planned in line with the results obtained.

The research findings indicate that strategy-based teaching improves students' formulation skills, as well as the cognitive and psychological processes involved in the learning process. Finding Relationships, Using Variables, Drawing Diagrams, and Simplification Strategies facilitated cognitive load management by helping students structure problem situations. When evaluated within the context of Cognitive Load Theory (Sweller, 1988), such structured teaching processes are thought to contribute to students forming more effective mental models for solving problems requiring higher-level thinking by reducing the load on their working memory. This explains the development observed in students' processes of converting real-life situations into mathematical structures.

Strategy-based problem-solving training can also be said to have significant effects on students' metacognitive awareness levels. Within the framework of the training, students' explanations of why they used specific strategies, their comparisons of alternative methods, and their discussions of the reasons for choosing a particular strategy enabled them to act more consciously in metacognitive processes such as planning, monitoring, and evaluation. These findings are consistent with studies emphasizing the positive effect of metacognitive awareness on problem-solving performance (Veenman et al., 2006; Dhlamini, 2016; Hartelt and Martens, 2024). Within the framework of strategy training, students developed thinking skills by reviewing and formulating their own thought processes, as well as reaching the correct conclusions. Furthermore, strategy-based applications are believed to have a positive impact on students' learning motivation and self-efficacy. By solving a complex problem step by step, taking Polya's steps into account, students have strengthened their perception of success and increased their intrinsic motivation to learn.

The primary starting point of this study is the classification of PS strategies in the context of mathematical process skills, as presented by Temel (2018). Temel (2018) categorized “Finding Relationships”, “Using Variables”, “Drawing Diagrams” and “Simplification” strategies within the formulating process in his classification of PS strategies. As a result of the analyses carried out within the scope of the research, it was found that “Finding Relationships”, “Using Variables”, “Drawing Diagrams” and “Simplification” strategies training improved students' formulating skills. This finding supports classification (Temel's, 2018). At the same time, Temel and Altun (2022) concluded that PS strategies improve students' ML skills. The study's results also coincide with those of Temel and Altun (2022).

In conclusion, this study is considered to have made significant contributions to the development of students' formulating processes and to the understanding of the underlying cognitive mechanisms of these processes. The findings reveal that the ability to formulate is not only a mathematical operation but is also closely related to higher-level mental processes, such as problem-solving, cognitive load management, problem representation, and metacognitive regulation. The strategy-based teaching process has positively contributed to the success of students in formulating solutions by developing their skills in identifying the given elements in a problem, examining relationships between situations, determining variables, and creating appropriate mathematical representations, while balancing cognitive load and increasing metacognitive awareness. The results obtained within the scope of the research reveal that considering theoretical structures, such as Cognitive Load Theory and Metacognitive Regulation, in teaching processes can be effective in developing formulating skills and improving students' problem-solving performance and mathematical literacy levels. Furthermore, from this perspective, the research is expected to contribute to the field of educational psychology both theoretically and practically by considering the interaction of cognitive and psychological processes in the development of formulating skills.

## 5 Limitations

The research was conducted within the framework of quantitative research methods and quantitative data collection techniques. Consequently, the findings limit the ability to provide a comprehensive reflection of contextual factors and detailed qualitative insights regarding participants' experiences.

The study was conducted with tenth-grade students studying in Anatolian high schools. This may limit the direct generalizability of the findings obtained to students studying in different school types (e.g., Science High Schools, Vocational High Schools, or Fine Arts High Schools) and to individuals at different grade levels. Additionally, the study was conducted with 52 students, 26 of whom were assigned to the experimental group and 26 to the control group. In future studies, a broader research population can be identified, and a more comprehensive understanding of formulating skills can be gained by conducting research with large sample groups, including students from all grade levels or different school types.

The research's implementation process took 5 weeks, with 3 weeks of training and 2 weeks of FT implementation. This time frame may limit the inferences about the long-term persistence of the improvements observed in students' formulating skills.

The study's data were obtained from the FT, which included 15 problems classified in the formulating dimension of PISA mathematical literacy problems. In future studies, research findings can be presented from a broader perspective, with problems developed to measure formulating skills and measurement tools consisting of more problems.

In conclusion, it is essential to consider the limitations mentioned above when evaluating the study's findings to gain a comprehensive perspective on the research. Future studies involving students from diverse school types and socioeconomic backgrounds, which examine the long-term permanence of skill development through comprehensive practices, including both qualitative and quantitative data collection methods, and present new findings in a detailed manner, will make significant contributions to the field.

## Data Availability

The original contributions presented in the study are included in the article/Supplementary material, further inquiries can be directed to the corresponding author.
